# Gut microbiota signatures and fecal metabolites in postmenopausal women with osteoporosis

**DOI:** 10.1186/s13099-023-00553-0

**Published:** 2023-07-06

**Authors:** Han Wang, Jing Liu, Zuoxing Wu, Yangyang Zhao, Man Cao, Baohong Shi, Baolong Chen, Ning Chen, Hao Guo, Na Li, Jian Chen, Ren Xu

**Affiliations:** 1 State Key Laboratory of Cellular Stress Biology, School of Medicine, Faculty of Medicine and Life Sciences, No. 4221 Xiang’an South Road, Xiang’an District, Xiamen, Fujian Province 361102 China; 2 Xiamen Key Laboratory of Regeneration Medicine, Fujian Provincial Key Laboratory of Organ and Tissue Regeneration, School of Medicine, The First Affiliated Hospital of Xiamen University-ICMRS Collaborating Center for Skeletal Stem Cells, Xiamen University, Xiamen, 361005 China; 3 Department of Rehabilitation, Zhongshan Hospital of Xiamen University, No.201-209 Hubinnan Road, Siming District, Xiamen, Fujian Province 361000 China; 4 Xiamen Treatgut Biotechnology Co., Ltd, Xiamen, Fujian Province 361001 China; 5 Department of Endocrinology, Zhongshan Hospital (Xiamen), Fudan University, Xiamen, 361000 China; 6 State Key Laboratory of Cellular Stress Biology, School of Life Sciences, Faculty of Medicine and Life Sciences, Xiamen University, Xiamen, 361102 China

**Keywords:** Postmenopausal women, Gut microbiota, Fecal metabolites, Osteoporosis

## Abstract

**Background:**

Women suffer from various distress and disturbances after menopause, including osteoporosis, a risk factor associated with multiple diseases. Altered gut microbiota has been implicated in postmenopausal osteoporosis. In this study, to understand gut microbiota signatures and fecal metabolite changes in postmenopausal women with osteoporosis, 108 postmenopausal women were recruited for intestinal microbiota and fecal metabolite detection. Among these participants, 98 patients, who met the inclusion criteria, were divided into postmenopausal osteoporosis (PMO) and non-postmenopausal osteoporosis (non-PMO) groups based on bone mineral density (BMD). The compositions of gut bacteria and fungi were examined by 16 S rRNA gene sequencing and ITS sequencing, respectively. Meanwhile, fecal metabolites were analyzed using liquid chromatography coupled with mass spectrometry (LC-MS).

**Results:**

We found that bacterial α-diversity and β-diversity were significantly altered in PMO compared to non-PMO patients. Interestingly, fungi composition showed larger changes, and the differences in β-diversity were more significant between PMO and non-PMO patients. Metabolomics analysis revealed that fecal metabolites, such as levulinic acid, N-Acetylneuraminic acid, and the corresponding signaling pathways were also changed significantly, especially in the alpha-Linolenic acid metabolism and selenocompound metabolism. The screened differential bacteria, fungi, and metabolites closely correlated with clinical findings between these two groups, for example, the bacterial genus, *Fusobacterium*, the fungal genus, *Devriesia*, and the metabolite, L-pipecolic acid, were significantly associated with BMD.

**Conclusions:**

Our findings indicated that there were remarkable changes in gut bacteria, fungi, and fecal metabolites in postmenopausal women, and such changes were notably correlated with patients’ BMD ​​and clinical findings. These correlations provide novel insights into the mechanism of PMO development, potential early diagnostic indicators, and new therapeutic approaches to improve bone health in postmenopausal women.

## Introduction

Osteoporosis is a metabolic bone disease that is characterized by the decreased bone mass per volume-unit. The low bone mass results in increased bone fragility and destroys microstructure, thereby reducing bone strength and increasing the risk of fractures at different sites [1, 2]. Osteoporotic fractures which are most common in elderly women (> 55 years old) and men (> 65 years old) can significantly increase bone disease-related morbidity and mortality [3]. The increased risk of osteoporosis and the following fragility fractures are the serious consequences as women grew older. About 10% of the global population suffer from osteoporosis, among which postmenopausal women over 50 years old account for 30% [4, 5]. At present, the commonly used anti-osteoporosis drugs in clinical practice have been limited by a number of factors including unobvious efficacy, long-term medication, allergic reaction, and mandibular osteonecrosis [4]. Therefore, early identification of postmenopausal women at risk for fracture and searching for safe and effective preventive intervention strategies to reduce the risk of fracture have important clinical significance.

The gut microbiome is primarily responsible for the balance and maintenance of the interaction between host and microorganisms, mainly including bacteria, fungi and viruses [6, 7]. In healthy individuals, the mutual regulation between intestinal flora and the host helps to maintain normal gastrointestinal function [7, 8]. Apart from preventing toxins from entering the peripheral circulation, a healthy gastrointestinal tract contributes to regulating the absorption of nutrients and water, and forming an intestinal barrier [9, 10]. Due to the existence of intestinal barrier, intestinal microorganisms can safely reside in the intestine. However, disruption of the intestinal ecosystem results in various digestive ailments such as ulcerative colitis and Crohn’s disease, as well as obesity, diabetes, immune system dysregulation, and osteoporosis-related metabolic diseases [9, 11]. Among gut microbiota, the proportionately low content of fungi accounts for 0.1% of total intestinal microbes. Although fungi comprise such a small percentage of intestinal flora, these microorganisms indeed affect the occurrence and development of multi-system diseases [12, 13]. However, until now no evidence had implicated a relationship between fungi and osteoporosis.

Accumulating evidence indicate[s] that the pathological process of osteoporosis is regulated by gut microbes [14]. Clinical studies have reported that the overproliferation of intestinal flora is associated with the decreased bone mineral density (BMD). Patients with enteric bacterial over-growth syndrome generally appear low bone mineral density and osteomalacia, high levels of pro-inflammatory factors such as TNF-α and IL-1, and increased activated osteoclasts [15]. Due to the important regulatory role of intestinal flora on metabolism, lack of intestinal flora at birth leads to many physiological and metabolic changes in the body, including the reduced absorption of calories, vitamins, and nutrients, and the delayed height, weight, and organ development [16]. Gut microbiota dysbiosis causes immaturity of immune, vascular, endocrine, intestinal, and nervous systems, all of which are involved in the regulation of bone mass [17, 18]. Low-dose penicillin induces a decrease in intestinal microbiota in prepubertal mice (21 days old), resulting in changes in intestinal microbiota metabolites and the abnormalities of intestinal immune [19], while antibiotic therapy-mediated intestinal microbiota depletion accelerates weight and bone growth [20]. Therefore, it follows that there is a close link between gut flora and bone loss. Importantly, there are great differences in the intestinal flora colonized in different regions and populations. The gut microbiome of postmenopausal women reveals an altered community dynamic, with co-presentations of osteoporosis and/or osteopenia diagnoses [21]. Evidence gathered on the gut microbiota-bone axis suggests *Prevotella histicola* was specifically able to prevent estrogen deficiency-induced bone loss [22]. A randomized controlled trial revealed that a bioavailable isoflavone and probiotic treatment can improve bone status and estrogen metabolism in postmenopausal osteopenic women [23]. Therefore, gut microbes are directly involved in the regulation of bone metabolism in postmenopausal osteoporosis. However, the specific mechanisms between gut microbiota, their fecal metabolites, and bone metabolism remains unclear.

In this study, the differential gut bacteria and fungi of intestinal microbiota and fecal metabolites were analyzed between postmenopausal osteoporosis (PMO) and non-postmenopausal osteoporosis (non-PMO) women using 16 S rRNA gene sequencing and ITS sequencing. The present study provides potential early diagnostic indicators to discriminate PMO and offers new strategies for treating osteoporosis.

## Results

### The difference of intestinal microorganisms between PMO and non-PMO patients

**Fig. 1:**
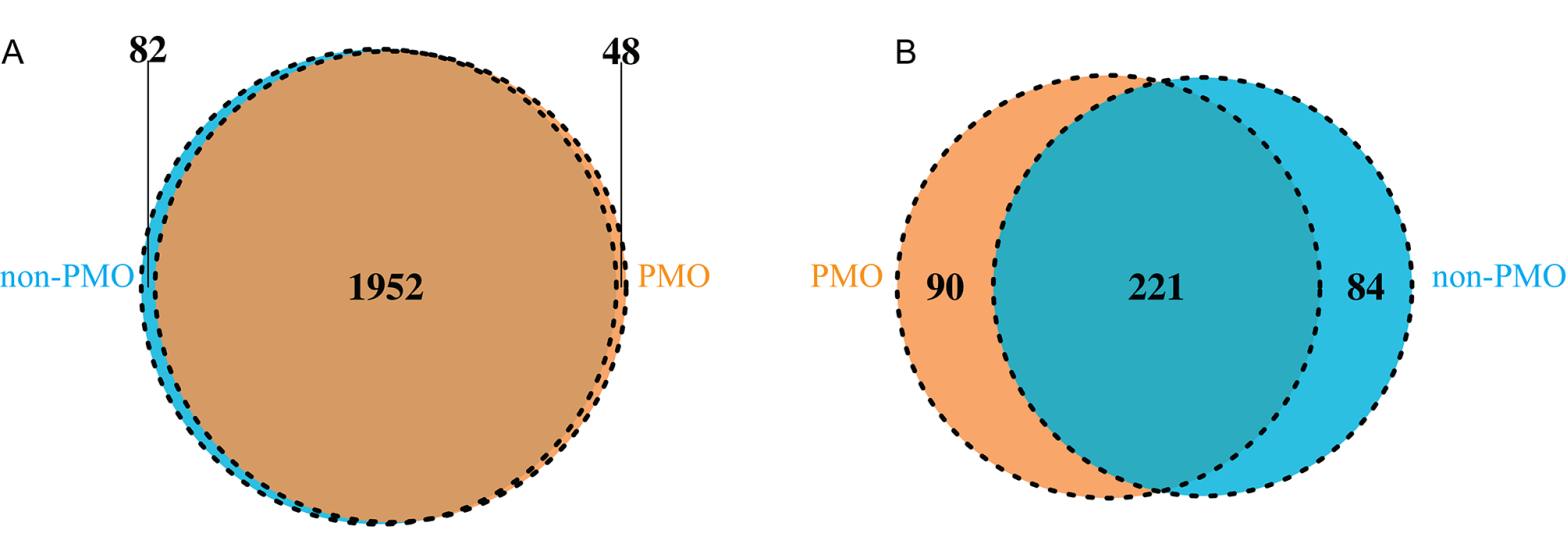
Venn diagram of gut microbiota composition in PMO and non-PMO patients. **(A)** Venn diagram demonstrating the differences in the distribution of gut bacteria between PMO and non-PMO populations. **(B)** Venn diagram demonstrating the differences in the distribution of gut fungi between PMO and non-PMO populations. PMO, postmenopausal osteoporosis; non-PMO, non-postmenopausal osteoporosis. Brown: the number of differential bacteria (n = 2000) or fungi (n = 2034) in PMO patients. Blue: the number of differential bacteria (n = 311) or fungi (n = 305) in non-PMO patients

To uncover the difference of intestinal microorganisms between PMO and non-PMO patients, we examined the abundance of gut bacteria and fungi in these two groups. Using 16 S rRNA sequencing, 82 exclusive bacteria genera were observed in non-PMO population, while 48 bacteria genera were screened in PMO cohort. Additionally, both groups shared 1,952 genera of bacteria **(**Fig. 1A**)**. In terms of fungi, 84 fungi were unique to the non-PMO population, while 90 fungi were unique to the PMO population, and 221 fungi were shared between these two groups **(**Fig. 1B**)**.

**Fig. 2:**
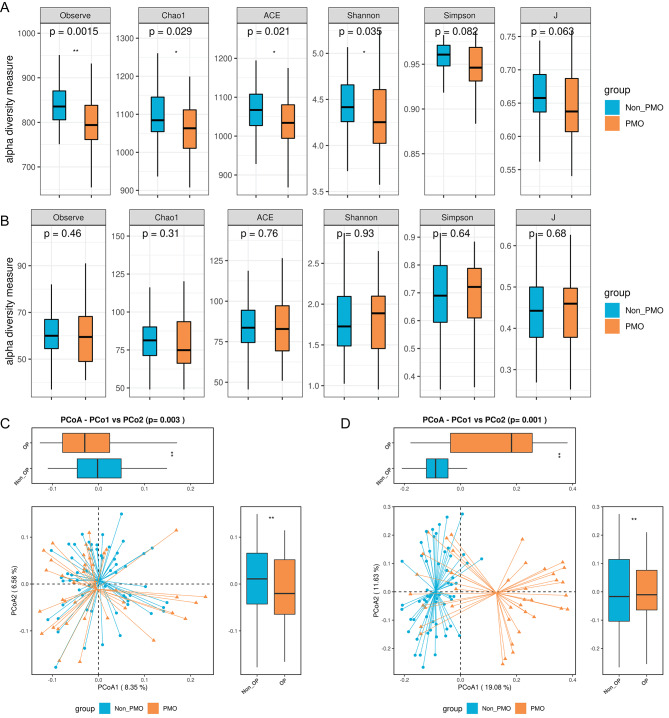
**Analysis of intestinal microbial diversity in PMO and non-PMO patients.** Box plot demonstrating α-diversity index, including Chao1 index, Abundance-based Coverage Estimator and Shannon index, Simpson index and J index in intestinal bacteria **(A)** and intestinal fungi **(B)** of PMO and non-PMO patients, repectively. Brown: PMO; Blue: non-PMO. PCoA plot was used to analyze the differential beta-diversity index in intestinal bacteria **(C)** and intestinal fungi **(D)** of PMO and non-PMO patients, repectively. *P < 0.05, **P < 0.01

Based on the in-depth analysis of gut bacteria, α-diversity index, Chao1 index, Abundance-based Coverage Estimator (ACE) and Shannon index were significantly decreased in PMO population compared to that in non-PMO patients **(**Fig. 2A**)**. In comparison to non-PMO, although Simpson index and J index showed a decreasing trend in PMO population, there was no significant difference between them. However, there was no significant difference in α-diversity index, Shannon index, and Simpson index of fungi between non-PMO population and PMO population **(**Fig. 2B**)**. β-diversity, which is an important index to evaluate the distribution of bacterial and fungal genera, showed a significant decrease in gut bacteria of PMO patients and a significant increase in gut fungi of PMO patients by contrast to non-PMO patients **(**Fig. 2C and D**)**. Therefore, intestinal microorganisms changed greatly in PMO patients.

### Bacterial genus with differential abundance between PMO and non-PMO patients

**Fig. 3:**
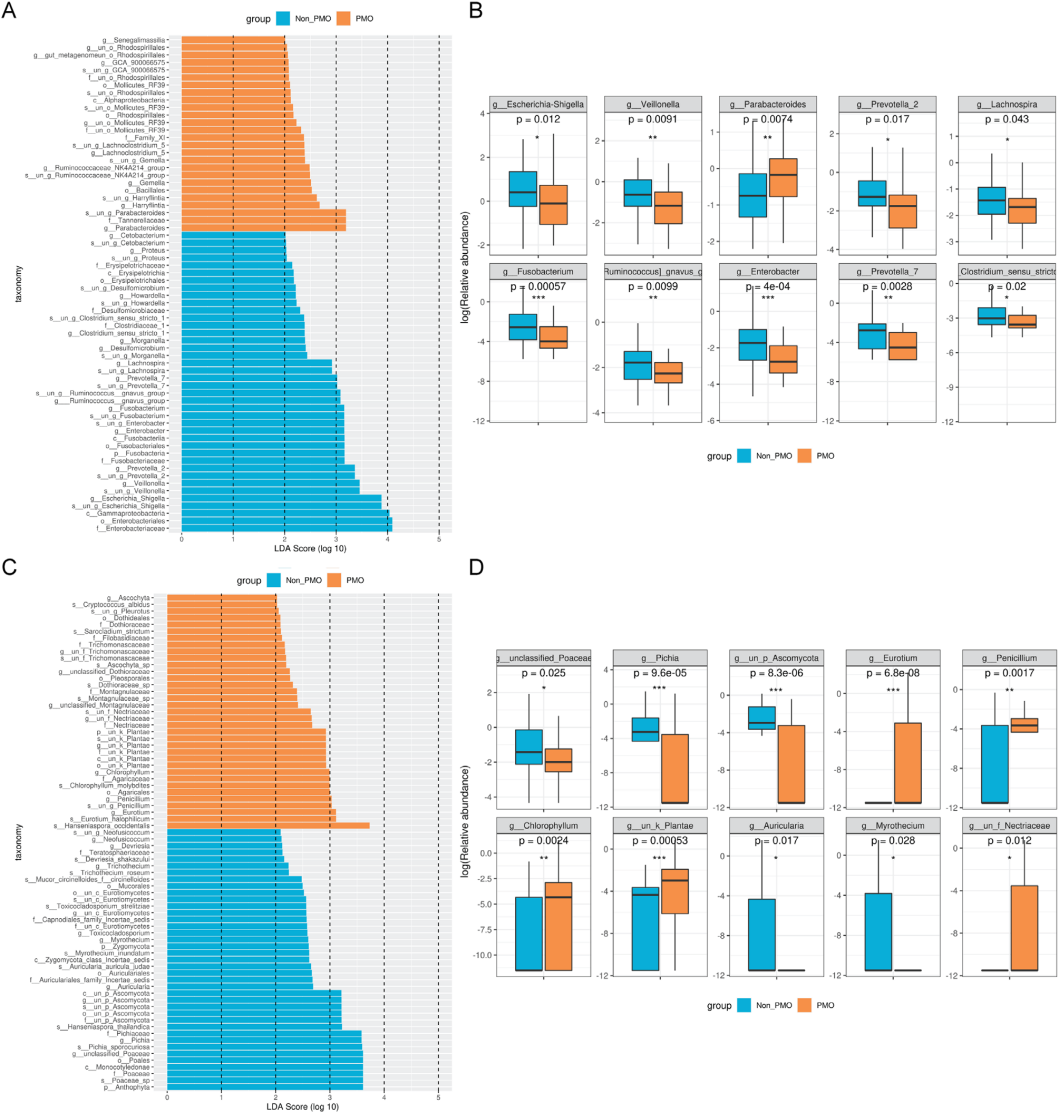
The differential bacterial community analysis between PMO and non-PMO patients. **(A and C)** Significantly enriched bacterial taxa **(A)** and fungi **(C)** in the different groups as determined by LEfSe analysis (LDA sore > 2). **(B and D)** Boxplot of the top 10 genera that showed the difference of the microbiota composition in bacteria **(B)** and fungi **(D)** level between PMO and Non-PMO groups. Brown: PMO; Blue: non-PMO. *P < 0.05, **P < 0.01, ***P < 0.001

The search for signature species between different populations contributes to exploring the distinct diagnostic factors and providing potential candidate targets for subsequent mechanism studies. The LDA Effect Size index was set as 2 between these two groups. Data indicated that *Veillonella*, *Parabacteroides*, and *Harryflintia* were mainly enriched in PMO population, while *Veillonella*, *Prevotella*, and *Enterobacterium* mainly appeared in non-PMO patients. Importantly, the abundance of these enriched bacteria differed significantly between the two groups **(**Fig. 3A and B**)**. In terms of fungi, *Pichia*, *Auricularia*, and *Myrothecium* were observed in non-PMO patients, while *Eurotium*, *Penicillium*, and *Chlorophyllum* were mainly concentrated in PMO population **(**Fig. 3C and D**)**. Thus, there were significant differences in genus levels between PMO and non-PMO patients.

### Analysis of intestinal microbial metabolic pathways in PMO and non-PMO patients

**Fig. 4:**
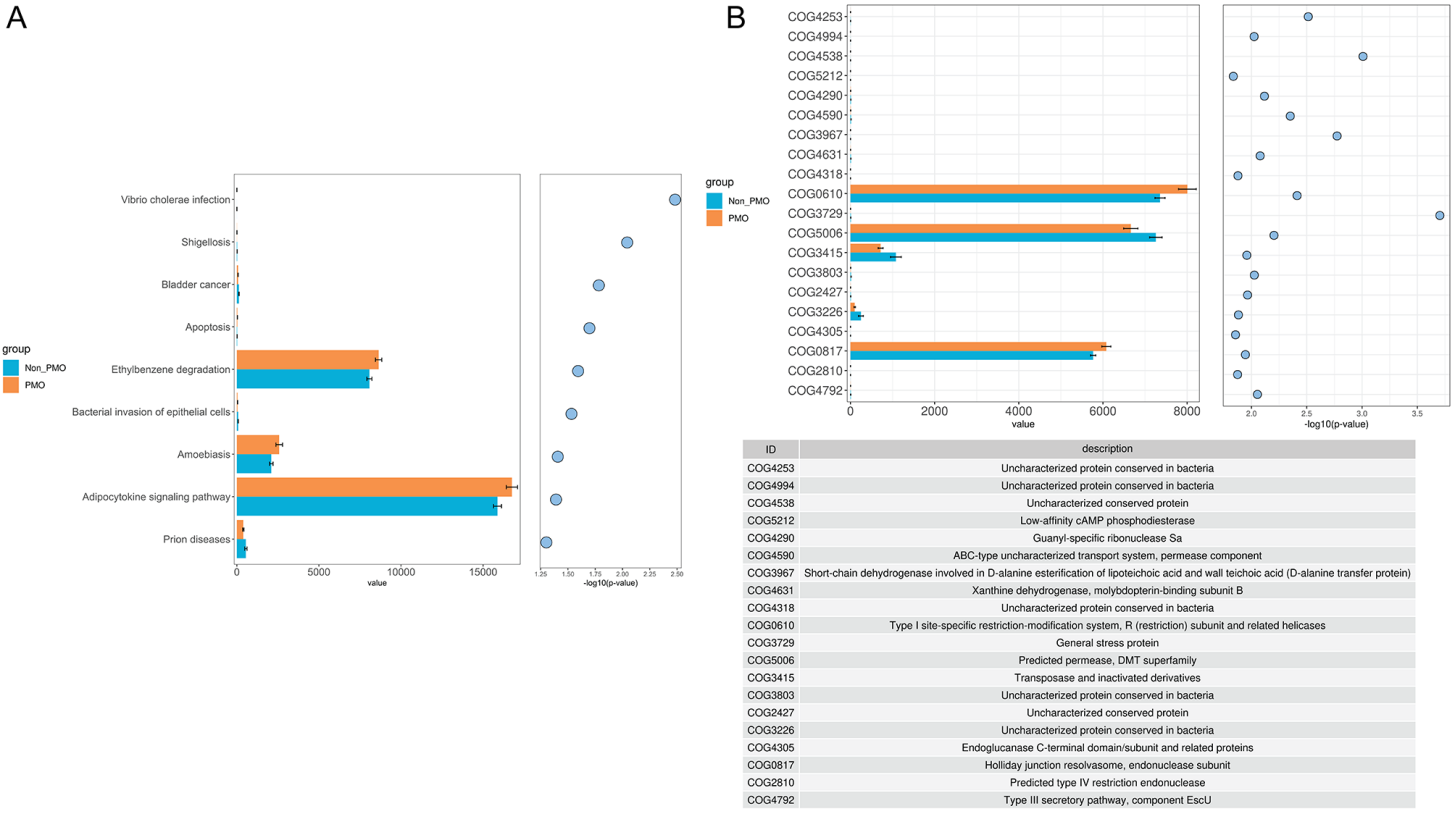
Metabolic pathways analysis between PMO and non-PMO patients. **(A)** KEGG was used to analyze the different metabolic pathways in intestinal bacteria. PICRUSt was used to calculate microbial abundance. Box plot demonstrating differential metabolomic pathways in intestinal bacteria of PMO and non-PMO women. **(B)** PICRUSt was used to calculate microbial abundance. COG was employed to analyze intestinal bacterial differential metabolic pathways. Box plot demonstrating differential metabolomic pathways (ID) of PMO and non-PMO women. Brown: PMO; Blue: non-PMO.

Metabolic pathway analysis was performed using the KEGG database. Based on KEGG database, PICRUSt analysis was applied to predict the functional profiling of microbial communities according to 16 S sequencing data. We confirmed that the enrichment of flora-related Adipocytokine signaling pathway, Amoebiasis pathway, and Ethylbenzene degradation pathway was significantly increased in PMO population compared with non-PMO population. However, the enrichment of pathways in bladder cancer, prion diseases, and bacterial invasion of epithelial cells were significantly decreased in PMO compared to non-PMO patients **(**Fig. 4A**)**. COG database was further used to analyze the top 20 potential metabolic pathways. The results indicated that the pathway related to transposase and inactivated derivatives was enriched in non-PMO population, and the pathway associated with Holliday junction resolvasome and endonuclease subunit were enriched in PMO patients **(**Fig. 4B**)**. Therefore, metabolic pathways were also changed significantly in PMO patients.

### Metabolomics analysis of fecal metabolites between PMO and non-PMO patients

**Fig. 5:**
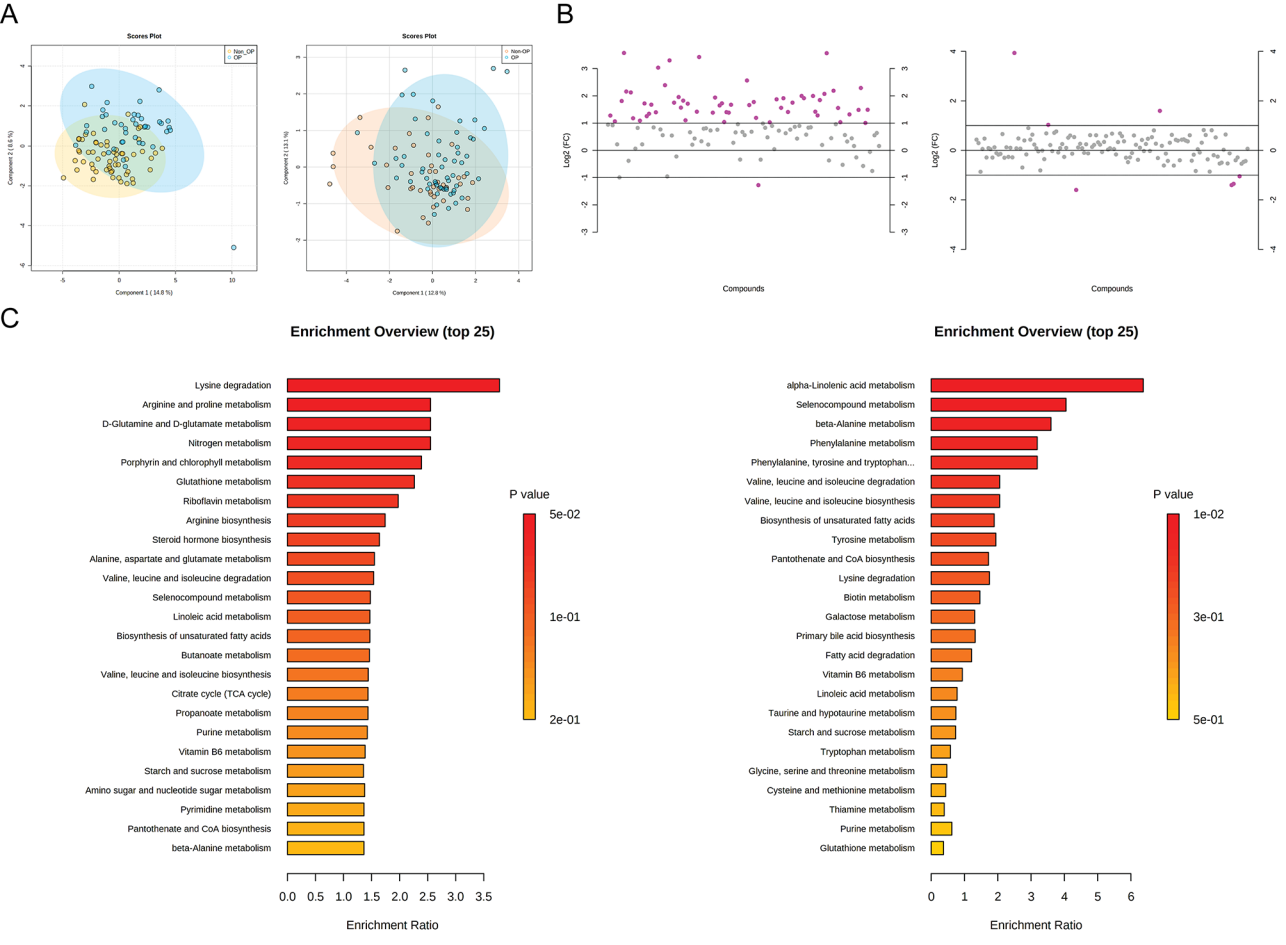
Metabolomics analysis of fecal metabolites between PMO and non-PMO patients. **(A)** Unsupervised principal component analysis (PCA) score plot demonstrating clear separation between fecal metabolomic profiles of PMO and non-PMO women based on all shared metabolites. PCA was calculated using MetaboAnalyst metabolomics analysis suite and ANOVA followed by Tukey’s multiple comparisons test using PRISM. **(B)** Scatter diagram demonstrating differential abundance of negative ion patterns (left) and positive ion patterns (right) of metabolomics between PMO and non-PMO patients. **(C)** Box plot demonstrating differential metabolite-related signaling pathways (top25) between PMO and non-PMO patients. PMO, postmenopausal osteoporosis; non-PMO, non-postmenopausal osteoporosis

To further evaluate the association between metabolites and bone mineral density, we employed metabolomics to detect the distribution of metabolites in these two groups. As shown in Fig. 5A, the principal component analysis (PCA) showed a significant difference in fecal metabolite compositions between non-PMO and PMO patients **(**Fig. 5A**)**. In brief, the abundances of Levulinic acid, N-Acetylneuraminic acid, Pimelic acid, Adenine, L-Lysine, Hydroxyisocaproic acid, and Azelaic acid notably raised in PMO population, while the abundance of Quinate, Kynurenic acid, Phit-val, Glycochenodeoxycholate, 1-Palmitoyl-sn-glycero-3-phosphocholine, and 1-Stearoyl-2-hydroxy-sn-glycero-3-phosphocho declined significantly in PMO patients, by contrast to that in non-PMO population **(**Fig. 5B**)**. Subsequently, cluster analysis revealed that the main enrichment pathways in PMO cohort included alpha-Linolenic acid metabolism, Selenocompound metabolism, beta-Alanine metabolism and Lysine degradation, Arginine and proline metabolism, D-Glutamine and D-glutamate metabolism and Nitrogen metabolism **(**Fig. 5C**)**. Possibly, the 16 S rRNA gene sequencing results showed that some components of the fecal microbiota significantly differed between the PMO and non-PMO groups; thus, we inferred that alterations in the fecal microbiota may lead to alterations in fecal metabolites.

### Association analysis of bacteria, fungi, and metabolites with clinical indicators in PMO and non-PMO patients

**Fig. 6:**
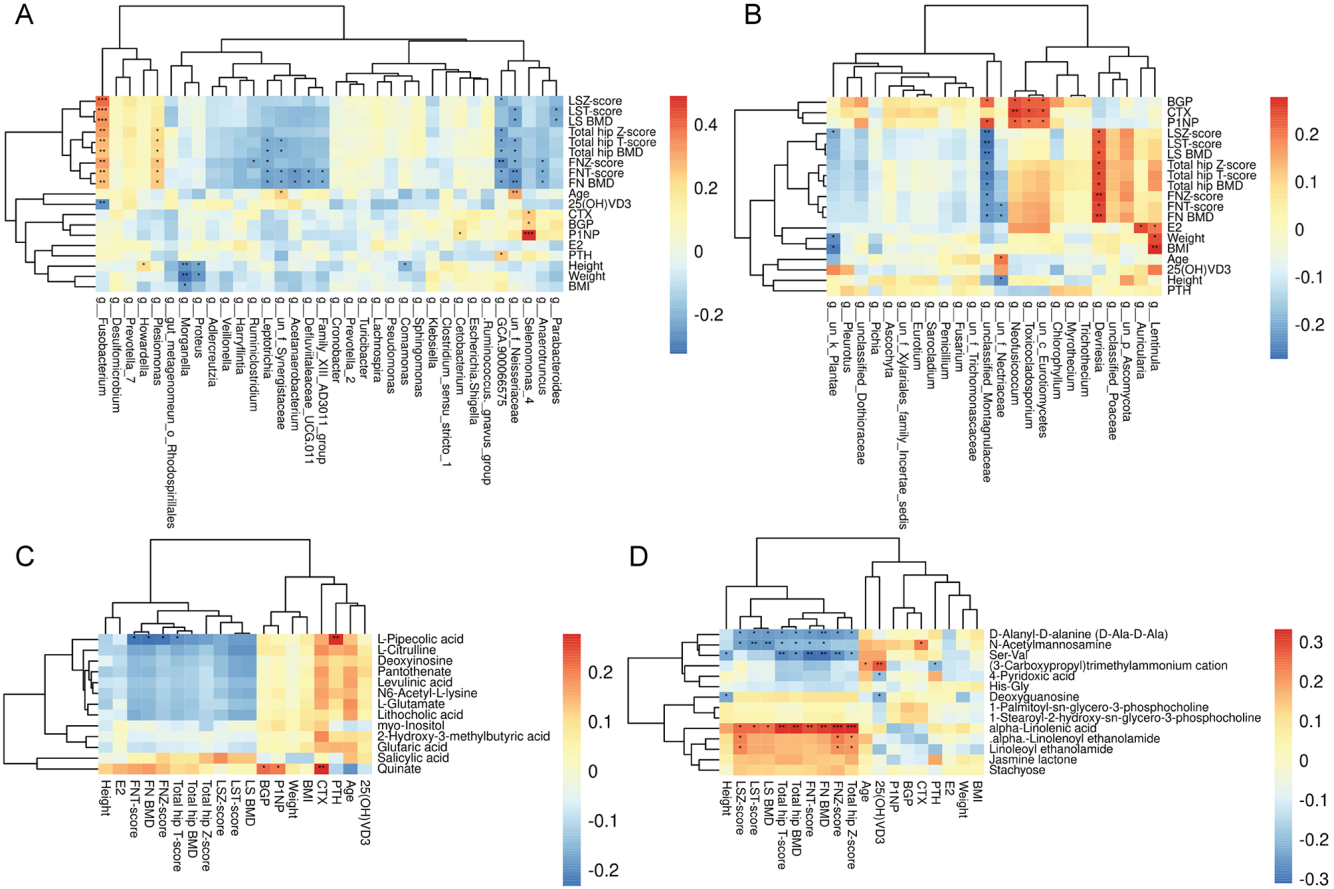
Clinical correlation analysis of bacteria, fungi, and metabolite in PMO-and non-PMO patients. **(A)** Clustering analysis demonstrating the correlation between intestinal bacterial and clinical indicators in PMO and non-PMO patients. **(B)** Clustering analysis demonstrating the correlation between the abundance of intestinal fungi and clinical indicators in PMO and non-PMO patients. **(C)** Clustering analysis demonstrating the correlation between metabolites and clinical indicators by mass spectrometry in the negative ion mode. **(D)** Clustering analysis demonstrating the correlation between metabolites and clinical indicators by mass spectrometry using positive ion-mode detection. PMO, postmenopausal osteoporosis; non-PMO, non-postmenopausal osteoporosis

Next, we analyzed the correlation between intestinal bacteria/fungi/metabolites and clinical indications using Pearson correlation analysis in PMO and non-PMO patients. Data revealed that gut bacterial *Fusobacterium*, *Parabacteroides*, *Anaerotruncus, Defluviitaleaceae, Acetanaerobacterium*, and *Leptotrichia* were closely related to BMD **(**Fig. 6A**)**. Gut fungal *Devriesia, Montagnulaceae*, and *Nectriaceae* were significantly correlated with BMD **(**Fig. 6B**)**. Metabolites related to BMD included L-Pipecolic acid, alpha-Linolenoyl ethanolamide, D-Alanyl-D-alanine(D-Ala-D-Ala), N-Acetylmannosamine, and Serine-Valine **(**Fig. 6C and D**)**. Thus, the altered intestinal bacteria/fungi/metabolites in PMO and non-PMO patients were related to BMD.

### Modeling analysis of PMO based on gut bacteria and fungi

**Fig. 7:**
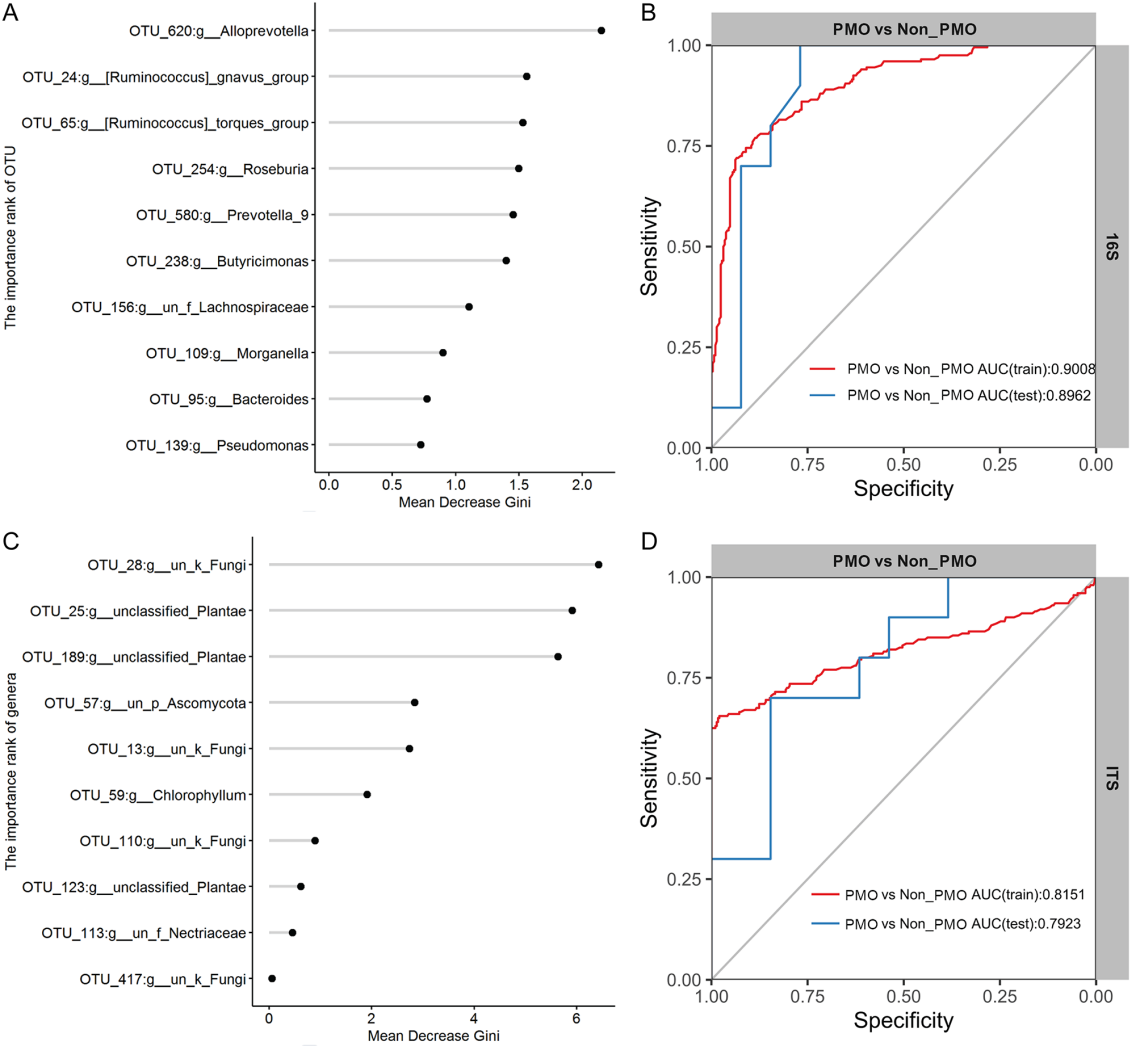
Intestinal microbiota-based model for distinguishing non-PMO patients from PMO patients. **(A)** Bacterial factors for intestinal bacteria-based PMO diagnosis model. **(B)** Line chart demonstrating the discriminant efficacy of the intestinal bacteria-based PMO diagnosis model in the exploration and validation cohorts. **(C)** Bacterial factors for intestinal fungi-based PMO diagnosis model. **(D)** Line chart demonstrating the differential efficacy of the intestinal fungi-based PMO diagnosis model in the exploration and validation cohorts. PMO, postmenopausal osteoporosis; non-PMO, non-postmenopausal osteoporosis

Subsequently, we established an identifying model for distinguishing non-PMO and PMO patients according to the differential bacterial and fungi communities. As shown in Fig. 7A, we developed a model using characteristic bacterial *Prevotella* as a classification factor according to the random forest model **(**Fig. 7A**)**. The results showed that the identification efficiency between PMO and non-PMO population was as high as 0.9008 **(**Fig. 7B**)**. Consistently, the discriminant efficiency reached 0.8151 when fungi were used as the classification factor in the model. To verify the true validity of our model, we further collected fecal samples from a prospective cohort of patients and performed fecal flora analysis. The discriminant efficiency of the bacteria-based classification model in this cohort was 0.8962, while the discriminant efficiency of the fungi-based classification model was 0.7923 **(**Fig. 7C and D**)**.

## Discussion

The intestinal tract is known as the second brain of human body, in which intestinal flora is a functional neuron that coordinates the operation of the whole-body system. Particularly, intestinal flora can participate in the regulation of postmenopausal osteoporosis [24, 25]. There is a close relationship between gut microbiome and bone turnover markers in postmenopausal women [26]. Numerous studies have explored the association between bone and intestinal flora in germ-free mice lacking intestinal flora, animal models treated with antibiotics or probiotics, and humans [27]. Although there are somewhat contradictions, the current consensus is that gut flora acts as a major regulator of BMD by influencing the immune system [28]. Supplementation with probiotics or a dietary fiber diet can regulate the distribution of intestinal flora, thereby reducing bone loss caused by estrogen loss [29, 30].

Gut microbiota changes have a significant impact on bone loss. There is growing evidence that connecting microbiome and menopause holds promise for new interventions to alleviate menopausal symptoms and for healthy ageing [31]. Lower gut microbiome diversity and a shift toward greater similarity to the male gut microbiome were confirmed in PMO patients [32]. Using 16 S rRNA gene sequencing, researchers have identified a close relationship between gut microbiota composition and osteoporosis/fracture risk in Japanese postmenopausal women [33]. However, there was no significant difference in bacterial α-diversity between the two groups. For example, there was no significant difference in bacterial α-diversity between the two groups. This is similar to an emerging report that confirmed the enrichment *Lactobacillus sp.* in non-PMO and the increased abundances of *Peptoniphilus* sp., propionic acid bacteria, and members of the *Galicola* genus in PMO [34]. Gut fungi accounts for a small proportion of intestinal microbes. However, it participates in the occurrence and development of multiple diseases [12, 13]. For instance, it has demonstrated that antifungals treatment can reduce liver damage in a fecal microbiome-humanized mouse model of Western diet-induced steatohepatitis [35]. Thus, fungi may play the important role in a variety of pathological processes. Our data were consistent with these findings. In the present study, species richness, diversity, and characteristic fungi appeared significantly different in PMO compared to that in non-PMO patients, indicating that there was a close correlation between fungi and osteoporosis. This finding further confirmed that fungi was an important regulatory factor among the overall biological flora in PMO patients. However, the detailed interaction between them is worth further study. In addition to that, our data proved a significant association between *Fusobacterium*/*Parabacteroides* with BMD of PMO patients. Consistently, previous studies have confirmed that the abundance of these bacteria was also altered in osteoporosis patients [36, 37]. In term of fungi, *Devriesia*, *Montagnulaceae* and *Nectriaceae* were associated with BMD indexes in this study. However, there is no evidence to confirm the association between these fungi species and osteoporosis. Thus, our data might provide a new target for exploring the mechanism underlying the process of PMO. Considering the positive association between *Devriesia* and BMD, and the negative association between *Montagnulaceae* and BMD, the two fungi species may play the beneficial and harmful roles during osteoporosis, respectively.

Several mechanisms underlie abnormal gut flora-mediated bone loss. Clinically, it has been found that the damaged strength in women’s spinal curvature strength is associated with a significant reduction in the gut microbiome, B cells, and T cells, which indicates a correlation between immune cell number and bone tissue characteristics [38]. Additionally, depletion of intestinal flora can lead to hyperimmune states [39]. Gut flora exerts lasting effects on the immune system either through direct contact or its metabolites. The increased bacterial abundance in the gut can raise the antigen load, leading to increased inflammatory cytokines, impaired osteoblast function, and bone loss in sickle cell disease (SCD) mice by compromising intestinal barrier of the immune system [40]. Previous study has indicated that menopause leads to increased gut permeability and inflammation, and greater gut permeability is related with more inflammation and lower bone mineral density across the menopause transition [41]. Thus, the association between gut flora and bone loss in PMO patients has the involvement of inflammation and immune regulation. In our study, significant changes in fecal metabolite abundance were observed in non-PMO and PMO populations, which may affect various metabolic pathways of the host, including alpha-Linolenic acid metabolism, etc. These metabolites and metabolic pathways have been shown to be closely related to bone metabolism and development [42, 43]. However, we also found that some novel metabolites, such as levulinic acid and pimelic acid, were significantly up-regulated in PMO population compared to non-PMO patients. Although no significant correlation was observed between these metabolites and BMD, these metabolites may still potentially influence the development of postmenopausal osteoporosis in women. Intestinal fungi are capable of developing symbiotic interactions with the hosts [12]. In brief, fungal wall components can be recognized by receptors of host cells, subsequently triggering antifungal signal transduction cascades and ultimately regulating innate and adaptive immune responses [44, 45]. Considering that intestinal bacteria-mediated the activation of immune system is closely related to the occurrence of osteoporosis, intestinal fungi may also regulate bone loss by regulating immune system.

The incidence of postmenopausal osteoporosis is about 57%. It is of great significance to effectively predict the occurrence of osteoporosis at the early stage [1, 46]. A recent study has reported 20 factors that closely affect osteoporosis. After screening with multiple feature selection methods, the differential efficiency of the random forest model is 0.921, which may be used as a practical method for the early diagnosis of postmenopausal osteoporosis [47]. Other studies have also proposed other potential predictors [48, 49]. In this study, we focused on the early diagnostic value of intestinal flora in PMO. Combined with the stochastic forest model, we confirmed that intestinal microecology including bacteria and fungi could act as a better identifier for predicting PMO. Importantly, although the efficacy of the validation cohort was reduced compared to the exploration cohort, the AUC was still higher than 0.8 for both bacteria and fungi.

## Conclusions

Collectively, our results suggested that postmenopausal women have significant changes in gut bacteria, fungi, and metabolites that are significantly correlated with BMD values and clinical features of PMO patients. This correlation provides potential directions for exploring the mechanism of PMO development and provides potential early diagnostic indicators for PMO. This study may provide novel interventions to improve the level of bone health in postmenopausal women.

## Materials and methods

### Study cohort

This study was a designed case-control study. This study was approved by the Ethics Committee of Zhongshan Hospital of Xiamen University (No. 201,808). Non-PMO subjects (n = 58) and newly diagnosed PMO patients (n = 40) who were admitted to Zhongshan Hospital Xiamen University from September 2021 up to now were recruited. Clinical information was presented in Table 1. For modeling analysis, a prospective cohort of non-PMO subjects (n = 10) and PMO patients (n = 13) were collected from Xinyu People’s Hospital (Jiangxi Province, CN). The trial number was ChiCTR1900027187. Clinical information of enrolled patients (modeling analysis) was presented in Table 2. Written informed consent was obtained from PMO patients and healthy people. The inclusion criteria were as follows: postmenopausal women were aged 50–70 years and were diagnosed with osteoporosis; All cohorts participated voluntarily in the study and signed the informed consent. The exclusion criteria were as below: people with organ dysfunction, neurological diseases (such as Parkinson’s syndrome, dementia, stroke, etc.), or rheumatic immune diseases were excluded; Patients with gastrointestinal diseases such as total parenteral nutrition, inflammatory bowel disease, and gastrointestinal surgery were also excluded from this study; patients with other serious diseases, such as malignant tumors or infectious diseases were not suitable for this study; patients who used antibiotics, other microecological preparations and gastrointestinal motility drugs that could affect intestinal flora within 30 days prior to enrollment. Patients who were taking or recently using Chinese and Western medicines such as calcitonin and zoledronic acid that could affect bone metabolism; patients with secondary osteoporosis were excluded; persons with mental or legal disabilities, additional patients unsuitable for inclusion and patients who are participating in other clinical trials were not suitable for the present research. The control group was postmenopausal non-osteoporosis patients (non-PMO). According to previous literature, the incidence of osteoporosis in postmenopausal women is 57%. Based on these, the effect size D1, α-value and β-value of difference in microflora between PMO and non-PMO populations were set as 0.57, 0.05, and 0.05, respectively. The expulsion rate was set as 5%. The sample size in the two groups was defined as 38 according to SPASS 15 software. Due to the 5% expulsion rate, 40 patients in each group were enrolled in this study.

**Table 1:** The clinicopathological factors of non-PMO (n = 58) patients and PMO (n = 40)

Characteristics	non-PMO	PMO	P-value
Age (year)	57.35 ± 3.98	59.69 ± 5.51	NS
BMI (kg/m^2^)	24.28 ± 2.79	23.8 ± 2.17	NS
LS BMD (g/cm^3^)	1.19 ± 0.11	0.8 ± 0.07	***
FN BMD (g/cm^3^)	0.98 ± 0.08	0.72 ± 0.1	***
Total hip	1.02 ± 0.09	0.76 ± 0.11	***
E2 (pmol/L)	45.85 ± 29.35	24.42 ± 7.47	***
25(OH)VD (ng/mL)	50.86 ± 17.7	56.28 ± 20.46	***
BGP (ng/mL)	19.96 ± 7.45	24.24 ± 13.25	NS
CTX-1 (ng/mL)	0.38 ± 0.18	0.48 ± 0.33	NS
P1NP (ng/mL)	54.92 ± 21.35	64.91 ± 43.46	NS
PTH (pg/mL)	45.4 ± 21.59	47.65 ± 26.08	NS

**Table 2:** The clinicopathological factors of non-PMO (n = 10) patients and PMO (n = 13)

Characteristics	non-PMO	PMO	P-value
Age (year)	57.5 ± 4.53	57.1 ± 6.01	NS
BMI (kg/m^2^)	21.79 ± 1.63	21.17 ± 2.06	NS
LS BMD (g/cm^3^)	0.98 ± 0.09	0.61 ± 0.07	***
FN BMD (g/cm^3^)	1.07 ± 0.14	0.78 ± 0.13	***
E2 (pmol/L)	42.2 ± 16.36	30.54 ± 17.22	NS
25(OH)VD (ng/mL)	45.95 ± 10.05	49.18 ± 10.91	NS
BGP (ng/mL)	15.06 ± 5.81	19.57 ± 15.34	NS
CTX-1 (ng/mL)	0.53 ± 0.21	0.7 ± 0.4	NS
P1NP (ng/mL)	45.67 ± 20.88	64.98 ± 69.18	NS

### Clinical data

Basic information such as age, height, and weight of all subjects were recorded, and body mass index (BMI) was calculated. Blood samples were collected for serological testing in the morning after more than 6-hour fasting. Serum levels of 25 (OH) D, estradiol (E2), osteocalcin (OC), C-terminal peptide of type I collagen (CTX-I), N-terminal propeptide of type 1 procollagen (P1NP), and parathyroid hormone (PTH) were measured using the Roche Diagnostics GmbH Electrochemical Luminescence System (Roche Diagnostics GmbH, Germany). Bone mineral density (BMD) in the lumbar spine (LS: L1-4) and total hip joint (femoral neck), trochanteric and intertrochanteric areas were measured using a diurnal calibrated Hologic 4500 dual-energy X-ray absorptiometry scanner (Lunar Expert 1313, Lunar Corp, USA).

### Fecal sample collection, DNA extraction, and 16 S rRNA gene sequencing

Stool samples from each volunteer were immediately stored in a -80 ℃ freezer after collection. After the stool samples were thawed and homogenized, total DNA was extracted from each sample (0.25 g) using the QIAamp Rapid DNA Stool Mini Kit (QIAGEN, Hilden, Germany) according to the manufacturer’s instructions. The concentration and purity of extracted DNA were measured by Multiskan™ GO full-wavelength enzyme marker (Thermo Fisher Scientific, US). DNA integrity was tested by agarose-gel electrophoresis. Bacterial and fungal communities were amplified by targeting the V4 region of 16 S rRNA gene and ITS2 fragment, respectively. The forward primer sequence of 16 S was 5’-GTGCCAGCMGCCGCGGTAA-3’, and the reverse primer sequence was 5’-GGACTACNVGGGTWTCTAAT-3’. The forward primer sequence of ITS2 was 5’-GCATCGATGAAGAACGCAGC-3’, and reverse primer sequence was 5’-TCCTCCGCTTATTGATATGC-3’. Polymerase chain reaction (PCR) products were purified and evaluated using Qubit 3.0 (Thermo Fisher Scientific, US). The PCR products were purified with Qiagen Gel Extraction Kit(Qiagen, Germany). Sequencing libraries were generated usingTruSeq® DNA PCR-Free Sample Preparation Kit (Illumina, USA) following manufacturer’s recommendations and index codes were added. The library quality was assessed on the Qubit@ 3.0 Fluorometer (Thermo Fisher Scientific, US) and Agilent Bioanalyzer 2100 system. At last, the library was sequenced on an Illumina NovaSeq platform and 250 bp paired-end reads were generated. Wilcoxon rank-sum test, that was set at a Monte-Carlo significance level α = 0.05 to calculate LDA scores, was used to detect features with different abundance levels between assigned taxa based on a normalized relative abundance matrix. All tests were performed using 999 permutations.

### Bioinformatics analysis

Paired-end reads were assembled using flash software. The primer sequences and the lower readings were removed. Checks for chimeric sequences and OTU clustering are performed using clean reads. All reads were demultiplexed into a single file, clustered with 97% similarity, and then the UNITE UCHIME reference dataset (version 7) was checked for Chimera examination using UCHIME in reference mode. The representative sequence was generated; the monomers were removed; and the final OTU table was created. Representative sequences of OTU were compared on the UNITE ITS database and classified by RDP classifier.

### Metabolomics

Twenty-five milligrams of fecal samples were mixed with 500 µL of extraction solution (methanol: acetonitrile: water = 2:2:1, internal standard mixture was labeled with isotope). Then, the samples were homogenized at 35 Hz for 4 min, followed by 5 min ultrasound in an ice water bath. The homogenization and ultrasound cycle were repeated for 3 times. After incubation at -40℃ for 1 h, the mixture was centrifuged at 4℃ at 12,000 rpm for 15 min, and the supernatant was transferred to fresh glass vials for subsequent analysis. Fecal metabolomics was measured using the Vanquish (Thermo Fisher Scientific) ultra-high performance liquid chromatograph. The target compounds were separated by Waters ACQUITY UPLC BEH Amide (2.1 mm × 100 mm, 1.7 μm) liquid chromatography column. Sample plate temperature maintained at 4℃ and sample volume was set as 2 µL. Mass spectrometry data was collected by the Orbitrap Exploris 120 mass spectrometer. Detailed parameters are as follows: sheath gas flow, 50 Arb; auxiliary gas flow, 15 Arb; capillary temperature, 320℃; full ms resolution, 60,000; MS/MS resolution,15,000; NCE mode collision energy, 10/30/60; spray voltage, 3.8 kV (positive) or -3.4 kV (negative).

### Functional analysis based on bacterial taxonomy

The unobserved State Reconstruction Community Phylogenetic Survey (PICRUSt) was used to predict metagenomic functional content. The sequencing data of 16 S was used to predict the presence of genes. First, the reference set GreenGenes database was read and a closed reference OTU table was constructed using QIIME software. The generated OTU table is normalized by copy number. Metagenomes were predicted using predict_metagenomes.py. ANOVA was used for statistical difference analysis. The results were visualized using a custom R script based on ggplot2.

### Machine learning models

Random Forest (R package and caret) models were trained on data from multiple omics analyses, including 16 S and ITS, respectively. The models were used to test whether data based on bacteria or fungi could predict osteoporosis. Firstly, the importance of common genera was ranked according to their average decline in accuracy. Next, we performed stepwise feature selection using a five-fold cross-validation approach to avoid over-fitting and over-optimistic estimates. This method is used to select and predict microbial characteristics and eliminate non-information characteristics. The area under ROC curve (AUC) was calculated to evaluate the differentiation of characteristic OTU.

### Statistical analysis and visualization

The estimation of α-diversity was based on a uniform and sparse OTU abundance matrix. Significant differences between α-diversity were examined using the nonparametric Kruskal-Wallis test and Benjamini Hochbery correction. The β-diversity that could estimate differences in community structure between samples was measured using the Bray-Curtis distance based on a uniform sparse OTU abundance table. Statistical differences in β-diversity measure were determined using R-package. OTU was calculated and visualized using the VennDiagram in R-package. Taxonomic abundance was measured and plotted using ggplot2. Different taxa abundance in different populations was monitored using LEfSe analysis. A genus-based index analysis was performed using R packaging to label species from genus information. Finally, a custom R script based on ggplot2 was used to visualize the results and the results were analyzed using R v3.4.1.

## Data Availability

The datasets used and/or analyzed during the current study are available from the corresponding author on reasonable request.

## List of abbreviations

PMO: postmenopausal osteoporosis
non-PMO: non-postmenopausal osteoporosis
BMD: bone mineral density
LC-MS: liquid chromatography coupled with mass spectrometry.
